# Advances in the Pharmacological Activities and Effects of Perilla Ketone and Isoegomaketone

**DOI:** 10.1155/2022/8809792

**Published:** 2022-10-28

**Authors:** Ruo Wang, Qianru Zhang, Chengling Feng, Juzhao Zhang, Yuxuan Qin, Linghua Meng

**Affiliations:** Shanghai Jiao Tong University School of Medicine, Shanghai 200025, China

## Abstract

As components of a traditional Chinese herbal medicine with many physiological activities, perilla ketone and isoegomaketone isolated from perilla essential oil are important active components of *Perilla frutescens*. Recent studies have shown that these two compounds have promising antitumor, antifungal, antirheumatoid arthritis, antiobesity, anti-inflammatory, healing-promoting, and other activities and can be used to combat toxicity from immunotherapy. Therefore, the multitude of pharmacological activities and effects demonstrate the broad research potential of perilla ketone and isoegomaketone. However, no reviews have been published related to the pharmacological activities or effects of perilla ketone and isoegomaketone. The purpose of this review is as follows: (1) outline the recent advances made in understanding the pharmacological activities of perilla ketone and isoegomaketone; (2) summarize their effects; and (3) discuss future research perspectives.

## 1. Introduction

Traditional Chinese medicines have a history of more than 2,000 years of use, but the real active ingredients and their mechanisms of action in most formulations are still unclear. Screening for active ingredients from traditional medicines is an important method for the discovery of new drugs [1, 2]. The 2015 Nobel Prize in Physiology or Medicine was awarded to a researcher for the discovery of the antimalarial drug artemisinin derived from traditional Chinese medicine, which is an example of success in the identification of the active molecules in these medicines [3, 4]. In summary, assessing the active molecules in Chinese medicinal materials is of great importance for the investigation and discovery of new drugs.

*Perilla frutescens* is an annual herb belonging to the genus Perilla, Lamiaceae. The main types of perilla are red (purple), green, and red/green perilla phenotypes with high polymorphism in morphology and taxonomy [5]. It is a traditional Chinese medicine with a history of more than 2000 years of use [6, 7]. According to ancient Chinese medicine texts, such as “Essential Prescriptions of the Golden Coffer,” *P. frutescens* has various effects, such as relieving the exterior and dispelling cold, invigorating qi and stomach, regulating qi and widening the middle, lowering qi, and eliminating phlegm [8]. That is, *P. frutescens* is considered to have the effects of treating cough and lung disease, influenza, fetal stress, seafood poisoning, etc. [9–11]. Modern pharmacological studies have also revealed that *P. frutescens* has antiallergy, anti-inflammatory, antioxidant, anticancer, antibacterial, antidepressant, and other activities [12–18]. To clarify the functions of the active components, many researchers have isolated and identified various compounds from *P. frutescens*, including flavonoids, volatile oils, fatty acids, triterpenes, and phenolic compounds [19–26].

*P. frutescens* volatile oil also contains a variety of active ingredients, such as perillaldehyde, limonene, myristin, elemene, perilla ketone, and isoegomaketone [27–33]. Among them, perilla ketone and isoegomaketone have been shown to exhibit beneficial drug activities in recent years [7, 34]. Recent studies have shown that these two compounds have promising antitumor, anti-inflammatory, antifungal, antirheumatoid arthritis, antiobesity, healing-promoting, and other activities and can be used to combat toxicity due to immunotherapy. Therefore, the multitude of pharmacological activities and effects of perilla ketone and isoegomaketone provide evidence of their broad research potential. However, no reviews have been published related to the pharmacological activities or effects of perilla ketone and isoegomaketone. The purpose of this review is as follows: (1) outline the recent advances made in the understanding of the pharmacological activities of perilla ketone and isoegomaketone; (2) summarize their effects; and (3) discuss future research perspectives.

## 2. Perilla Ketone

**Figure 1:**
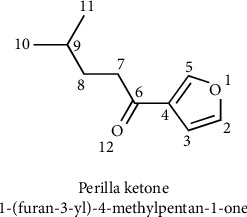
The chemical structure of perilla ketone.

Perilla ketone (1-(furan-3-yl)-4-methylpentan-1-one) is an important component of the volatile oil of *P. frutescens*; the structure is shown in Figure 1, and it mainly exists in *P. frutescens* leaves [35, 36]. Sebe reported for the first time that perilla ketone was an important component in the volatile oil of *P. frutescens* [37]. Afterward, the chemical synthesis of perilla ketone was studied. Matsuura was the first to report on the synthesis of perilla ketone via 3-furanoyl chloride and organocadmium reagents [38]. Farina et al. reported that the Stille reaction could be applied to the preparation of perilla ketone with a yield of 74% [39]. In a study of the biosynthetic pathway of perilla ketone, Ito et al. reported for the first time that perilla ketone is mainly based on the secondary metabolite produced by isopentenyl diphosphate via the mevalonate pathway, which is synthesized by a selective addition reaction [40–42].

Early studies on perilla ketone focused on the toxicity of perilla ketone in animals; specifically, perilla ketone induces interstitial pneumonia, severe respiratory distress, and death in animals, such as cattle and sheep. The mechanism of action may be that perilla ketone increases pulmonary microvascular permeability and thus induces toxicity [43, 44]. To date, there has been insufficient evidence to determine whether perilla ketone can induce similar toxic effects in humans, which may be related to differences in the monooxygenase system [45–47]. In recent years, with the increase in research on perilla ketone, it was found to have antitumor, antifungal, anti-inflammatory, and other activities and to be able to combat toxicity due to immunotherapy in addition to its toxic effects in animals, suggesting that perilla ketone has the potential to be developed as an active drug molecule.

### 2.1. Antitumor Activity

The antitumor activity associated with perilla ketone was investigated in vitro by Chen et al. Perilla ketone was shown to have similar activity to paclitaxel against human gastric adenocarcinoma MGC-803 cells and human nonsmall cell lung cancer A549 cells (IC50: 17.82 ± 5.12 *μ*g/mL∼21.31 ± 0.98 *μ*g/mL). Notably, the effects of other compounds derived from *P. frutescens* and perilla ketone may synergize to enhance its antitumor efficacy [35].

### 2.2. Combatting Toxicity due to Immunotherapy

Adoptive T-cell therapy is a promising method for tumor immunotherapy [48–50], but there are potential challenges, such as inflammatory factor storms and unpredictable off-target and organ-specific toxicity [51–53]. The potential toxicity associated with adoptive T-cell therapy can be well controlled by the induction of apoptosis in genetically modified T cells by small molecule drugs [54, 55]. CYP4B1 is a mammalian cytochrome P450 monooxygenase [54, 55]. In adoptive T-cell therapy, CYP4B1 can induce apoptosis through some small molecules, such as 4-ipomeanol or perilla ketone, but the specific mechanism is still unclear [54, 55]. Linette et al. conducted an in vitro study on the apoptosis-inducing activity of perilla ketone in adoptive T-cell therapy and found that the use of only 2.9 mmol·L^−1^ perilla ketone resulted in the death of 84 ± 1.08% of ΔNGFR-T2A-CYP4B1P +12-transduced T cells with little toxicity to primary T cells that were not transduced. In this study, perilla ketone was 3–5 times more active than the positive control drug 4-ipomeanol and induced apoptosis in transduced cells at a faster rate and at lower concentrations [52]. A similar study was conducted by Morgan et al. [53]. These studies suggest that perilla ketone has the potential to prevent the toxicity associated with adoptive T-cell therapy [54, 56].

Thesseling et al. conducted a computational simulation study on the mechanism of adoptive T-cell apoptosis induced by perilla ketone and 4-ipomeanol, and the results showed that under the action of the CYP4B1 enzyme, the furan functional group of 4-ipomeanol was activated by epoxidation. Under the action of the CYP4B1 enzyme, perilla ketone can undergo two chemical reactions: epoxidation of the furan functional group or hydroxylation of the isopropyl functional group. Either reaction can promote the activation of perilla ketone. The difference in the mechanism of CYP4B1 enzyme action on perilla ketone and on 4-ipomeanol may explain why perilla ketone has a greater ability to induce apoptosis than the positive control drug, 4-ipomeanol [57].

### 2.3. Antifungal Activity

The antifungal effect of perilla ketone was investigated by Prole and Taylor The results showed that perilla ketone could affect biofilm formation in various fungi, including *C. musae*, *F. dimerum,* and *F. oxysporum* and reduce conidia adhesion and germination and the development of structural biofilms [58, 59]. Further research on the mechanism of action revealed that perilla ketone exerts antifungal activity by activating the highly conserved transient receptor potential (TRP) channel and affecting the surface sensing mechanism of fungi [58, 59].

### 2.4. Anti-Inflammatory Activity

Wang et al. conducted in vitro experiments on the anti-inflammatory effect of perilla ketone. The results showed that perilla ketone has a good ability to prevent the effects of inflammatory mediators, such as NO, TNF-*α,* and/or IL-6, in vitro in lipopolysaccharide-stimulated mouse monocyte-macrophage RAW264.7 cells [60].

### 2.5. Other Activities

TRP ion channels are channel proteins that are widely distributed in the peripheral and central nervous systems and have broad research prospects in the fields of nociception, sensory perception, and tumor therapy targets [61–64]. Bassoli et al. tested the in vitro TRPA1 agonistic activity of perilla ketone using rTRPA1 expressed in HEK293 human embryonic kidney cells. The results showed that perilla ketone exhibited good TRPA1 agonistic activity in vitro. Only approximately 80.7 ± 1.6 *μ*M of isoegomaketone was needed to promote the agonistic activity of the control drug, which was 100 *μ*mol·L^−1^ allyl isothiocyanate [65].

## 3. Isoegomaketone

**Figure 2:**
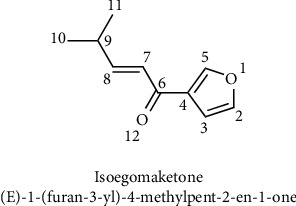
The chemical structure of isoegomaketone.

Isoegomaketone ((E)-1-(furan-3-yl)-4-methylpent-2-en-1-one), the structure of which is shown in Figure 2, is a newly-discovered compound derived from *P. frutescens*. The volatile components were first isolated and identified by Baser et al. with gas chromatography–mass spectrometry (GC–MS) [66]. Regarding its biosynthesis, Tabata et al. reported for the first time that the C14 isotope labeling method indicated that the hypothesis put forth by Hegnauer and Fujita, which was that the biosynthesis of isoegomaketone does not involve the use of isohexanone as a precursor but is controlled by the inhibitory gene I3, with beracenone double bond isomerization synthesis performed for the precursor [67–69]. During the past decade, research on the activity of isoegomaketone has gradually increased, especially regarding its antitumor activity and anti-inflammatory activity, which have been widely reported.

**Figure 3:**
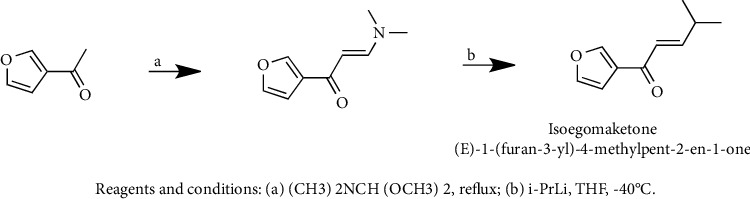
Chemical synthesis of isoegomaketone [70].

In addition to natural biosynthetic mechanisms, the chemical synthesis of isoegomaketone has also been reported. Park et al. reported that the synthesis of isoegomaketone achieved an approximately 31% yield through a simple two-step reaction using 1-(furan-3-yl)ethan-1-one as a substrate [70]. The specific reaction process is shown in Figure 3.

### 3.1. Antitumor Activity

#### 3.1.1. Colorectal Cancer

The anticancer activity of isoegomaketone in the context of colorectal cancer was investigated in vitro by CHO et al. The results showed that isoegomaketone exhibited activity against DLD1 human colorectal adenocarcinoma cells in a dose-dependent manner in the range of 10–100 *μ*mol·L^−1^, with an IC_50_ of approximately 25 *μ*mol·L^−1^. Further mechanistic studies showed that isoegomaketone could activate cytochrome c in the mitochondria by promoting the cleavage of poly ADP ribose polymerase; enhancing the activity of the apoptosis-related enzymes caspase-8, -9, and -3; and promoting the cleavage of Bid and the translocation of Bax. This finding revealed that isoegomaketone induces apoptosis in DLD1 human colorectal cancer cells through the mitochondria-dependent cytochrome c pathway [71, 72]. In addition, isoegomaketone can induce apoptosis in DLD1 human colorectal cancer cells in a mitochondrial AIF-dependent pathway by inducing the translocation of the apoptosis-inducing factor AIF from the inner and outer intermembrane space of the mitochondria into the nucleus [73, 74].

**Figure 4:**
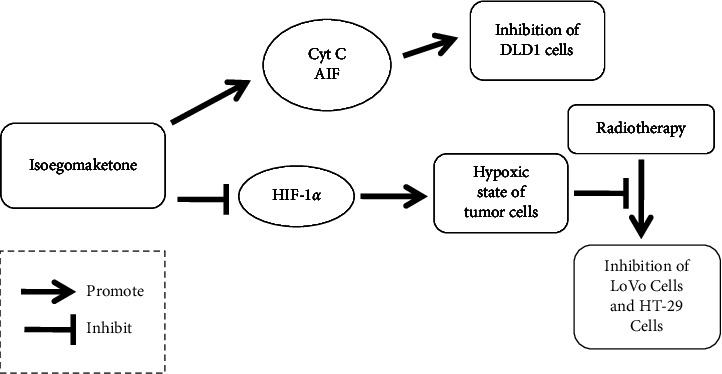
Possible effects of isoegomaketone-mediated inhibition of colorectal cancer cells.

Wu et al. conducted an in vivo experiment investigating the combined use of isoegomaketone and ionizing radiation in the treatment of colorectal cancer. Nude mice loaded with human colorectal cancer LoVo cell xenografts and human colorectal cancer HT-29 cell xenografts were shown to be significantly different from each other. After three weeks of treatment, the solid tumors in the group subjected to combined isoegomaketone administration and ionizing radiation shrank significantly more (solid tumor volume: 1.133 ± 0.115 cm^3^, 1.267 ± 0.252 cm^3^) than those in the group subjected to ionizing radiation alone (1.500 ± 0.100 cm^3^, 1.800 ± 0.200 cm^3^), but the survival rate in the combined treatment group was lower than that in the radiotherapy group. The reason is unclear, and more research is needed. Research on the mechanism of action has shown that isoegomaketone can improve the hypoxic state of tumor cells by inhibiting the expression of HIF-1*α*, thereby increasing the sensitivity of colorectal cancer cells to radiation and enhancing the effect of radiotherapy [75]. The possible effects of isoegomaketone-mediated inhibition of colorectal cancer cells are shown in Figure 4.

#### 3.1.2. Melanoma

The antimelanoma activity of isoegomaketone was studied by Kwon et al. The results of cell experiments showed that isoegomaketone inhibited mouse melanoma B16 cells (IC50 approximately 50–100 *μ*mol·L^−1^), and the activity was dose-dependent in the range of 25–100 *μ*mol·L^−1^. In addition, the researchers observed that at approximately 50 *μ*mol·L^−1^, isoegomaketone could affect the number, shape, and adhesion of colorectal cancer cells. In in vivo experiments in mice, administration of isoegomaketone at 5 mg–20 mg/kg significantly inhibited the increase in melanoma volume. Mechanistic studies have shown that isoegomaketone induces the generation of ROS [76], which in turn activates the mitochondria-dependent CytC and AIF pathways, ultimately leading to apoptosis [77].

**Figure 5:**
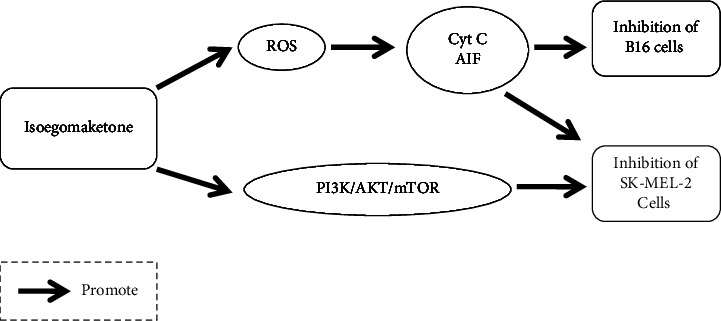
Possible effects of isoegomaketone-mediated inhibition of melanoma cells.

Seo et al. also conducted in vitro experiments and mechanistic studies on the antimelanoma activity of isoegomaketone. Their results showed that isoegomaketone also significantly inhibited the growth of SK-MEL-2 human skin malignant melanoma cells in a dose-dependent manner with an IC_50_ of approximately 25–50 *μ*mol·L^−1^. Its mechanism of action was similar to that previously reported by Kwon et al. In addition, in SK-MEL-2 cells treated with isoegomaketone, the researchers observed significant reductions in p-AKT and p-mTOR levels. This finding revealed that isoegomaketone can inhibit the growth of melanoma cells by affecting the upstream PI3K/AKT/mTOR signaling pathway in addition to inducing ROS generation and then activating mitochondria-dependent apoptosis pathways, such as those involving cytochrome c and AIF, to exert antimelanoma activity [78]. The possible effects of isoegomaketone-mediated inhibition of melanoma cells are shown in Figure 5.

#### 3.1.3. Lung Cancer

Yang et al. studied a combination of isoegomaketone and ionizing radiation for the treatment of lung cancer. The results of the in vitro study showed that after 48 hours of treatment, the inhibition rate achieved using isoegomaketone combined with ionizing radiation in human nonsmall cell lung cancer A549 cells reached a maximum of 59.2%, which was significantly better than the inhibition rate achieved using ionizing radiation alone (up to approximately 26.9%). Mechanistic studies have shown that isoegomaketone increases the sensitivity of human lung cancer cells to radiation by affecting the levels of the endoplasmic reticulum stress proteins IREL, ATF6, and PERK, thereby enhancing the effect of radiotherapy. In addition, treatment with isoegomaketone combined with ionizing radiation can affect the levels of the apoptosis-related proteins COX-2, CDX-2, *β*-catenin, and E-cadherin and can promote the expression of Bcl2, VEGF, and PCNA. These elevated protein levels may also be associated with radiosensitization, but this possibility requires further study [79].

#### 3.1.4. Prostate Cancer

**Figure 6:**
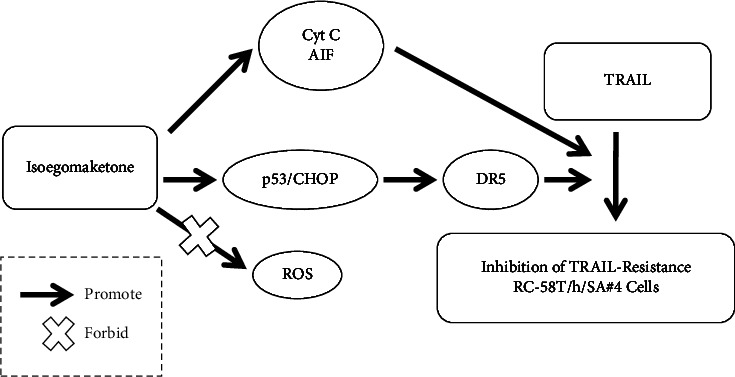
Possible effects of isoegomaketone-mediated inhibition of prostate cancer cells.

TNF-related apoptosis-inducing ligand (TRAIL) is a tumor-selective molecule that can transmit death signals to cells by binding to a receptor (TRAIL-R1). TRAIL has received extensive attention due to its highly selective proapoptotic effect and because it is nontoxic in normal cells, and it is considered to be a promising antitumor drug [80, 81]. However, the resistance of some tumor cells to TRAIL makes the development of TRAIL sensitizers urgent [82]. Lee et al. conducted in vitro experiments and mechanistic studies on the therapeutic activity of isoegomaketone in combination with TRAIL. The results showed that the combined use of isoegomaketone and TRAIL inhibited the viability of TRAIL-resistant RC-58T/h/SA#4 primary malignant human prostate cancer cells by 80%, and their combined use showed little toxicity in normal RWPE-1 human prostate cells. Mechanistic studies have shown that isoegomaketone sensitizes cells to TRAIL-mediated apoptosis through the mitochondria-dependent cytochrome c pathway, mitochondria-dependent AIF pathway, and death receptor DR5 pathway. In addition, the levels of p53 and CHOP were increased, suggesting that p53 and CHOP are involved in inducing sensitization. The sensitizing effect of isoegomaketone was confirmed to be independent of ROS, which is different from the common antitumor mechanism of isoegomaketone, suggesting that further research is needed [83]. The possible effects of isoegomaketone-mediated inhibition of prostate cancer cells are shown in Figure 6.

#### 3.1.5. Liver Cancer

In Wanga et al.'s study on the antihepatoma activity of isoegomaketone, it was found that it could significantly inhibit the activity of human hepatoma cells (HCCs). Mechanistic studies have shown that isoegomaketone exerts anticancer effects by blocking the PI3K/Akt signaling pathway [84].

Wu et al. also conducted extensive cellular-level studies on the activity of isoegomaketone against HCC. The results showed that isoegomaketone could significantly (*p* < 0.05) inhibit the growth and proliferation of HepG2, Huh7, and Huh7-HBx human hepatoma cells, and the cell proliferation activity after treatment with isoegomaketone was approximately 39–46% that in the control group. In addition, Wu et al. studied the radiotherapy effect of isoegetomone combined with ionizing radiation on Huh7 and Huh7-HBx cells. The results showed that isoegomaketone significantly increased the radiosensitivity (*p* < 0.05), and the effect of the combined treatment was approximately the same as that using ionizing radiation alone and was 1.9–2.4 times than that in the control group.

Mechanistic studies have shown that isoegomaketone exerts its anti-inflammatory effect mainly by increasing the mRNA expression levels of the inhibitory proliferators p15, p18, p21, p27, and p53; increasing the levels of proapoptotic proteins Fas, TRAILR1, and Bax; and decreasing the levels of the proliferation activators Bcl-2 and FLIP. When used with methods for radiosensitization, it has a synergistic sensitizing effect on antitumor activity [85].

### 3.2. Anti-Inflammatory Activity

Park et al. used a synthetic isoegomaketone compound to study its in vitro anti-inflammatory activity in mouse monocyte-macrophage RAW 264.7 cells. The results showed that isoegomaketone had high activity against the inflammatory factors NO, MCP-1, and IL-6. Mechanistic studies have shown that isoegomaketone regulates gene transcription mediated by NF-*κ*B and AP-1 by reducing their transcriptional activity [70].

Jin et al. conducted animal experiments and mechanistic studies investigating the anti-inflammatory activity of isoegomaketone. Isoegomaketone was administered by injection to BALB/c mice injected with lipopolysaccharide. The results showed that both the NO level and iNOS protein level in the serum of the mice were reduced, suggesting the anti-inflammatory activity of isoegomaketone in vivo. Researchers have explored its mechanism of action at the cellular level. In lipopolysaccharide-induced RAW 264.7 macrophages, TLR4 is activated, which promotes the activation of the TRIF-dependent pathway, increases the level of iNOS inflammatory factors, and produces a proinflammatory effect. Isoegomaketone, however, can inhibit TRIF-dependent pathways and inhibit INF-*β*-INFR-mediated STAT-1 phosphorylation, ultimately decreasing iNOS levels, and thereby exerting anti-inflammatory activity. In addition, isoegomaketone can promote the translocation of Nrf2 to the nucleus, thereby inducing increased HO-1 expression, which in turn inhibits STAT-1 phosphorylation, decreases iNOS levels, and exerts anti-inflammatory effects [86].

In contrast to previous studies, Jin et al. reported a different anti-inflammatory mechanism underlying the effects of isoegomaketone, arguing that isoegomaketone achieves its anti-inflammatory effect mainly by inhibiting the IFN-*β*-STAT-1 pathway rather than the NF-*κ*B-AP-1 pathway. Inflammation and weak inhibition of the NF-*κ*B-AP-1 pathway may inhibit the activation of TRAF6 or RIP1 through TRIF [86].

**Figure 7:**
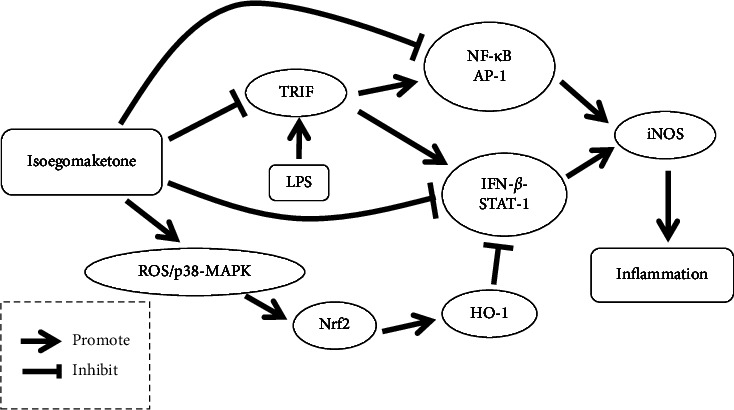
Possible effects of isoegomaketone-mediated inhibition of inflammation [86].

Jin studied the mechanism by which isoegomaketone promotes the translocation of Nrf2 to the nucleus and induces HO-1 expression. Isoegomaketone-induced HO-1 mRNA expression was only inhibited by a specific inhibitor of p38-MAPK, while ROS scavengers also blocked isoegomaketone-induced ROS production and HO-1 expression. The above experiments demonstrate that isoegomaketone induces HO-1 expression via the ROS/p38-MAPK/Nrf2 pathway in RAW264.7 cells. Interestingly, this study revealed that isoegomaketone induces the production of other antioxidant enzymes, such as CAT, NQO-1, and GST, in addition to HO-1. These antioxidant enzymes have been confirmed to have anti-inflammatory activities, suggesting that the anti-inflammatory mechanism of isoegomaketone remains to be further clarified [87]. The possible effects of isoegomaketone-mediated inhibition of inflammation are shown in Figure 7.

### 3.3. Healing-Promoting Activity

Kim et al. conducted in vitro experiments and mechanistic studies on the healing-promoting activity of isoegomaketone. The results showed that isoegomaketone significantly (*p* < 0.05) promoted the proliferation and migration of human keratinocyte HaCaT cells. The proliferation level and migration area of HaCaT cells treated with 10 *μ*mol·L^−1^ isoegomaketone for 24 hours were approximately 1.5 times those in the control group. Mechanistic studies have shown that isoegomaketone promotes cell proliferation by activating the MAPK/ERK pathway [88].

### 3.4. Antirheumatoid Arthritis Activity

Chang et al. conducted animal experiments on the antirheumatoid arthritis effect of isoegomaketone. The results showed that after oral administration of isoegomaketone at 10 mg/kg/day for 7 days in collagen antibody-treated BALB/c mice, the arthritis symptoms of the mice were significantly (*p* < 0.05) attenuated. Hind paw swelling and redness were scored at approximately 30% of the level observed in the control group. Histopathological studies showed that the degree of inflammatory cell infiltration and the degree of edema formation in the ankle joints of the treated mice was lower in the treatment group than in the control group. Additionally, the neutrophil-to-lymphocyte ratio (NLR) in the whole blood of mice in the treatment group was reduced by approximately 85% that in the control group [89].

### 3.5. Antiobesity Activity

The antiobesity effect of isoegomaketone was investigated by So et al. First, in cellular experiments, isoegomaketone inhibited differentiation of and lipid accumulation in 3T3-L1 cells. Mechanistic studies have shown that isoegomaketone significantly inhibits the mRNA expression of adipocyte-specific genes associated with 3T3-L1 cell differentiation. Later, in an in vivo experiment using 45% high-fat diet-induced C57BL/6J obese mice, it was found that when they were given isoegomaketone at 10 mg/kg/day, the increase in body weight and the increase in visceral fat in the mice were only approximately 70% and 50% of the control group values, respectively [90].

## 4. Future Research Perspectives

Although perilla ketone and isoegomaketone show promising pharmacological activities and there is preliminary data regarding their mechanisms, they still require further study.

### 4.1. Toxicity

As mentioned earlier, the toxicity of perilla ketone in animals has been reported, but the toxicity of isoegomaketone has not. Additionally, perilla ketone and isoegomaketone have not been reported to exhibit similar toxicity in humans. To date, the toxicity of perilla ketone and isoegomaketone has been slightly mentioned in studies on their pharmacological activities [83, 91]. Current toxicity studies have focused on mixtures such as perilla ketone and isoegomaketone-containing essential oils [92–94]. But these studies have not indicated the responsible compounds of toxicity. Safety studies on pure compounds of perilla ketone or isoegomaketone are still lacking [95–97]. This gap may limit the continued development of perilla ketone and isoegomaketone, although further research is expected.

### 4.2. Clinical Studies

Many reports on perilla ketone and isoegomaketone and their pharmacological activities and mechanisms have been described at a level in vitro, but unfortunately, the studies in vivo have been rare even further clinical studies [98]. And there has been no clinical research conducted from an evidence-based medicine perspective to support their clinical application. In fact, it is necessary to carry out a series of clinical studies on perilla ketone and isoegomaketone after sufficient safety assessments have been performed and more comprehensive investigations of their drug properties have been conducted.

### 4.3. Others

In addition to their associated toxicity and the lack of clinical studies, limitations in this area include that in silico studies and pharmacokinetic studies related to perilla ketone and isoegomaketone have not been reported. Indeed, in silico studies can provide prospective guidance on the chemical modification of compounds, while pharmacokinetic studies can provide kinetic data necessary for clinical trials. The absence of in silico studies and pharmacokinetic studies related to perilla ketone and isoegomaketone may limit their further chemical modification and in vivo applications.

## 5. Conclusion

In conclusion, perilla ketone and isoegomaketone isolated from traditional Chinese medicine have shown promising antitumor, anti-inflammatory, antifungal, antirheumatoid arthritis, antiobesity, and healing-promoting activities and have been shown to combat toxicity due to immunotherapy. Although great progress has been made in the prevention and treatment of cancer in recent years, insensitivity to radiotherapy and the side effects of immunotherapy are major obstacles to effective treatment. Perilla ketone can play a role in alleviating the potential toxicity associated with immunotherapy, and isoegomaketone can effectively promote the sensitivity of various tumor types to radiotherapy; thus, these compounds could have benefits when applied in combination with tumor radiotherapy and immunotherapy. In addition, related studies suggest that perilla ketone and isoegomaketone have corresponding antitumor effects, but up-to-date cytotoxicity and pharmacokinetic data are limited. In conclusion, perilla ketone and isoegomaketone can be used as candidates for the development of new drugs and are worthy of further development.

## Data Availability

No data were used to support the findings of this study. Figures were drawn and edited with ChemDraw 19.0 and Microsoft Office PowerPoint 2007 by R. W.
